# *catena*-Poly[[[aqua­[2-(6-chloro­pyridin-3-yl)acetato-κ*O*]sodium]-di-μ-aqua] monohydrate]

**DOI:** 10.1107/S1600536812014092

**Published:** 2012-04-13

**Authors:** Yuwei Mi, Dezhi Sun, Suyuan Zeng, Nana Yan

**Affiliations:** a College of Chemistry and Chemical Engineering, Liaocheng University, Shandong 252059, People’s Republic of China

## Abstract

The crystal structure of the title compound, {[Na(C_7_H_5_ClNO_2_)(H_2_O)_3_]·H_2_O}_*n*_, features polymeric chains along [010]. The Na^+^ cation is octa­hedrally coordinated by four bridging water mol­ecules, a terminal water mol­ecule and an O atom derived from a monodentate carboxyl­ate ligand. Adjacent polyhedra share two O⋯O edges. The polymeric chains are linked into a three-dimensional network *via* O—H⋯O and O—H⋯N hydrogen bonds.

## Related literature

For a related structure, see: Guo *et al.* (2004 ▶).
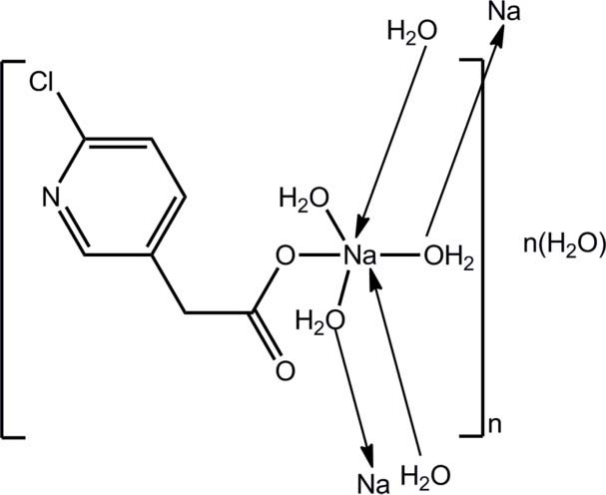


## Experimental

### 

#### Crystal data

[Na(C_7_H_5_ClNO_2_)(H_2_O)_3_]·H_2_O
*M* *_r_* = 265.62
Monoclinic, 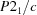

*a* = 12.4695 (12) Å
*b* = 5.5377 (5) Å
*c* = 17.0557 (17) Å
β = 91.190 (1)°
*V* = 1177.48 (19) Å^3^
*Z* = 4
Mo *K*α radiation
μ = 0.37 mm^−1^
*T* = 298 K
0.47 × 0.21 × 0.10 mm

#### Data collection

Siemens SMART CCD area-detector diffractometer
Absorption correction: multi-scan (*SADABS*; Sheldrick, 1996 ▶) *T*_min_ = 0.844, *T*_max_ = 0.964
5621 measured reflections
2082 independent reflections
1558 reflections with *I* > 2σ(*I*)
*R*_int_ = 0.027

#### Refinement

*R*[*F* ^2^ > 2σ(*F* ^2^)] = 0.032
*wR*(*F* ^2^) = 0.084
*S* = 1.05
2082 reflections
145 parameters
H-atom parameters constrained
Δρ_max_ = 0.19 e Å^−3^
Δρ_min_ = −0.21 e Å^−3^

### 

Data collection: *SMART* (Siemens, 1996 ▶); cell refinement: *SAINT* (Siemens, 1996 ▶); data reduction: *SAINT*; program(s) used to solve structure: *SHELXS97* (Sheldrick, 2008 ▶); program(s) used to refine structure: *SHELXL97* (Sheldrick, 2008 ▶); molecular graphics: *SHELXTL* (Sheldrick, 2008 ▶); software used to prepare material for publication: *SHELXTL*.

## Supplementary Material

Crystal structure: contains datablock(s) I, global. DOI: 10.1107/S1600536812014092/tk5075sup1.cif

Structure factors: contains datablock(s) I. DOI: 10.1107/S1600536812014092/tk5075Isup2.hkl

Additional supplementary materials:  crystallographic information; 3D view; checkCIF report

## Figures and Tables

**Table 1:** Selected bond lengths (Å)

Na1—O1	2.3632 (15)
Na1—O5^i^	2.3872 (16)
Na1—O3^ii^	2.4032 (16)
Na1—O4	2.4239 (17)
Na1—O3	2.5142 (17)
Na1—O5	2.5187 (16)

Symmetry codes: (i) 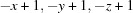; (ii) 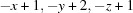.

**Table 2:** Hydrogen-bond geometry (Å, °)

*D*—H⋯*A*	*D*—H	H⋯*A*	*D*⋯*A*	*D*—H⋯*A*
O3—H3*A*⋯O4^iii^	0.85	2.06	2.893 (2)	168
O4—H4*A*⋯O2^iv^	0.85	1.91	2.762 (2)	175
O3—H3*B*⋯O6^v^	0.85	1.97	2.775 (2)	159
O4—H4*B*⋯O2^v^	0.85	1.98	2.824 (2)	169
O5—H5*A*⋯O6^v^	0.85	2.08	2.886 (2)	157
O5—H5*B*⋯O1^ii^	0.85	2.07	2.9214 (19)	175
O6—H6*A*⋯N1^vi^	0.85	2.05	2.900 (2)	173
O6—H6*B*⋯O2	0.85	1.95	2.796 (2)	176

Symmetry codes: (ii) 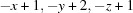; (iii) 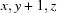; (iv) 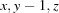; (v) 
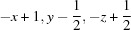
; (vi) 
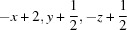
.

**Fig. 1.**
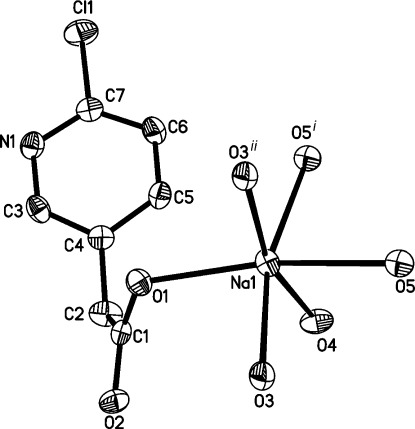
The coordination environment around the Na atom showing numbering scheme. Displacement ellipsoids are drawn at the 30% probability level. Symmetry codes: i - x + 1, -y + 1, -z + 1; ii - x + 1, -y + 2, -z + 1. The lattice water molecule and hydrogen atoms have been omitted.

**Fig. 2.**
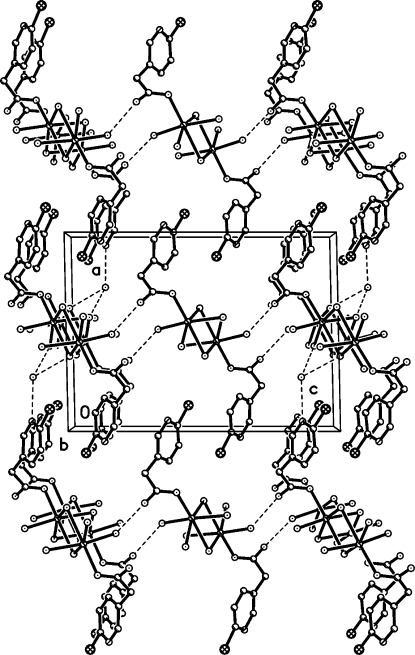
Packing diagram of (I), showing hydrogen bonds as dashed lines.

## supplementary crystallographic information

### Comment

In the title compound (Fig. 1), (I), the Na atom has a distorted O_6_ octahedral environment formed by a terminal water molecule O4, four O atoms derived from µ_2_-bridging water molecules and one carboxylate (O1) atom of the 6-chloro-3-pyridineacetate ligand (Table 1). Each coordination octahedron shares two O···O edges with two adjacent octahedra, thus producing infinite chains. Similar chains were found in the structure of sodium carboxynitrobenzoate tetrahydrate (Guo, 2004). In (I), the chains are linked into a three-dimensional network *via* O—H···O and O—H···N hydrogen bonds (Table 2 and Fig. 2).

### Experimental

To a solution of 6-chloro-3-pyridineacetic acid (1 mmol) in doubly-distilled water (60 ml ), a solution of an equimolar amount of sodium hydroxide in doubly-distilled water (40 ml) was added drop wise at room temperature. After vigorous stirring for 4 h, the resulting mixture was evaporated *in vacuo* to a volume of about 20 ml and filtered hot. The filtrate was then set aside for crystallization at room temperature. Two weeks later, colourless crystals of the title compound were isolated.

### Refinement

The H atoms were placed in geometrically idealized positions (O—H = 0.85, C—H = 0.93–0.97 Å) and treated as riding on their parent atoms, with *U*_iso_(H) = 1.5*U*_eq_(O) or *U*_iso_(H) = 1.2*U*_eq_(C).

### Crystal data

**Table:**

[Na(C_7_H_5_ClNO_2_)(H_2_O)_3_]·H_2_O	*F*(000) = 552
*M**_r_* = 265.62	*D*_x_ = 1.498 Mg m^−^^3^
Monoclinic, *P*2_1_/*c*	Mo *K*α radiation, λ = 0.71073 Å
Hall symbol: -P 2ybc	Cell parameters from 5621 reflections
*a* = 12.4695 (12) Å	θ = 2.4–27.1°
*b* = 5.5377 (5) Å	µ = 0.37 mm^−^^1^
*c* = 17.0557 (17) Å	*T* = 298 K
β = 91.190 (1)°	Block, colourless
*V* = 1177.48 (19) Å^3^	0.47 × 0.21 × 0.10 mm
*Z* = 4	

### Data collection

**Table:**

Siemens SMART CCD area-detector diffractometer	2082 independent reflections
Radiation source: fine-focus sealed tube	1558 reflections with *I* > 2σ(*I*)
Graphite monochromator	*R*_int_ = 0.027
φ and ω scans	θ_max_ = 25.0°, θ_min_ = 1.6°
Absorption correction: multi-scan (*SADABS*; Sheldrick, 1996)	*h* = −9→14
*T*_min_ = 0.844, *T*_max_ = 0.964	*k* = −6→6
5621 measured reflections	*l* = −19→20

### Refinement

**Table:**

Refinement on *F*^2^	Primary atom site location: structure-invariant direct methods
Least-squares matrix: full	Secondary atom site location: difference Fourier map
*R*[*F*^2^ > 2σ(*F*^2^)] = 0.032	Hydrogen site location: inferred from neighbouring sites
*wR*(*F*^2^) = 0.084	H-atom parameters constrained
*S* = 1.05	*w* = 1/[σ^2^(*F*_o_^2^) + (0.0308*P*)^2^ + 0.4882*P*] where *P* = (*F*_o_^2^ + 2*F*_c_^2^)/3
2082 reflections	(Δ/σ)_max_ = 0.001
145 parameters	Δρ_max_ = 0.19 e Å^−^^3^
0 restraints	Δρ_min_ = −0.21 e Å^−^^3^

### Special details

**Table:**

Geometry. All e.s.d.'s (except the e.s.d. in the dihedral angle between two l.s. planes) are estimated using the full covariance matrix. The cell e.s.d.'s are taken into account individually in the estimation of e.s.d.'s in distances, angles and torsion angles; correlations between e.s.d.'s in cell parameters are only used when they are defined by crystal symmetry. An approximate (isotropic) treatment of cell e.s.d.'s is used for estimating e.s.d.'s involving l.s. planes.
Refinement. Refinement of *F*^2^ against ALL reflections. The weighted *R*-factor *wR* and goodness of fit *S* are based on *F*^2^, conventional *R*-factors *R* are based on *F*, with *F* set to zero for negative *F*^2^. The threshold expression of *F*^2^ > σ(*F*^2^) is used only for calculating *R*-factors(gt) *etc*. and is not relevant to the choice of reflections for refinement. *R*-factors based on *F*^2^ are statistically about twice as large as those based on *F*, and *R*- factors based on ALL data will be even larger.

### Fractional atomic coordinates and isotropic or equivalent isotropic displacement parameters (Å^2^)

**Table:**

	*x*	*y*	*z*	*U*_iso_*/*U*_eq_	
O6	0.73121 (12)	1.2862 (3)	0.13436 (9)	0.0499 (4)	
Na1	0.55064 (6)	0.74749 (14)	0.44657 (5)	0.0350 (2)	
Cl1	1.11649 (5)	0.25311 (12)	0.44137 (4)	0.0589 (2)	
N1	1.04684 (13)	0.6295 (3)	0.36393 (11)	0.0399 (5)	
O1	0.69179 (11)	0.9744 (3)	0.39281 (8)	0.0363 (4)	
O2	0.64644 (11)	1.1400 (3)	0.27723 (8)	0.0395 (4)	
O3	0.42626 (11)	1.0983 (3)	0.42246 (8)	0.0412 (4)	
H3A	0.4419	1.2194	0.3943	0.049*	
H3B	0.3694	1.0368	0.4027	0.049*	
O4	0.51265 (12)	0.4951 (3)	0.33326 (9)	0.0453 (4)	
H4A	0.5559	0.3922	0.3147	0.054*	
H4B	0.4714	0.5431	0.2961	0.054*	
O5	0.37654 (11)	0.6269 (2)	0.50779 (8)	0.0381 (4)	
H5A	0.3354	0.6360	0.4675	0.046*	
H5B	0.3590	0.7388	0.5391	0.046*	
H6A	0.7978	1.2532	0.1339	0.046*	
H6B	0.7082	1.2413	0.1786	0.046*	
C1	0.70075 (15)	0.9966 (4)	0.32056 (12)	0.0311 (5)	
C2	0.78312 (18)	0.8466 (4)	0.27717 (13)	0.0461 (6)	
H2A	0.7443	0.7362	0.2428	0.055*	
H2B	0.8230	0.9552	0.2440	0.055*	
C3	0.97016 (17)	0.7608 (4)	0.32733 (13)	0.0398 (5)	
H3	0.9909	0.9016	0.3021	0.048*	
C4	0.86306 (16)	0.7010 (4)	0.32460 (12)	0.0337 (5)	
C5	0.83412 (18)	0.4939 (4)	0.36460 (13)	0.0395 (5)	
H5	0.7623	0.4490	0.3662	0.047*	
C6	0.91043 (18)	0.3544 (4)	0.40187 (13)	0.0428 (6)	
H6	0.8919	0.2143	0.4284	0.051*	
C7	1.01530 (17)	0.4294 (4)	0.39851 (12)	0.0368 (5)	

### Atomic displacement parameters (Å^2^)

**Table:**

	*U*^11^	*U*^22^	*U*^33^	*U*^12^	*U*^13^	*U*^23^
O6	0.0327 (9)	0.0665 (11)	0.0504 (10)	0.0073 (8)	0.0012 (7)	0.0212 (8)
Na1	0.0387 (5)	0.0315 (4)	0.0349 (5)	−0.0025 (4)	0.0060 (4)	0.0001 (4)
Cl1	0.0542 (4)	0.0584 (4)	0.0637 (4)	0.0218 (3)	−0.0098 (3)	−0.0013 (3)
N1	0.0308 (10)	0.0401 (11)	0.0491 (11)	0.0015 (9)	0.0052 (9)	−0.0046 (9)
O1	0.0378 (8)	0.0405 (8)	0.0310 (8)	−0.0005 (7)	0.0078 (6)	0.0007 (7)
O2	0.0328 (8)	0.0476 (9)	0.0378 (9)	0.0095 (7)	−0.0022 (7)	−0.0004 (8)
O3	0.0423 (9)	0.0397 (9)	0.0413 (9)	−0.0108 (7)	−0.0040 (7)	0.0033 (7)
O4	0.0487 (9)	0.0488 (9)	0.0382 (9)	0.0147 (8)	−0.0060 (7)	−0.0062 (7)
O5	0.0420 (9)	0.0361 (8)	0.0361 (8)	0.0018 (7)	−0.0001 (7)	−0.0013 (7)
C1	0.0249 (10)	0.0327 (11)	0.0357 (12)	−0.0057 (9)	0.0008 (9)	−0.0015 (10)
C2	0.0482 (14)	0.0550 (14)	0.0352 (12)	0.0189 (12)	0.0073 (10)	−0.0003 (11)
C3	0.0409 (13)	0.0340 (11)	0.0449 (13)	0.0021 (10)	0.0143 (10)	0.0043 (11)
C4	0.0349 (12)	0.0371 (12)	0.0294 (11)	0.0074 (9)	0.0065 (9)	−0.0050 (9)
C5	0.0321 (12)	0.0382 (12)	0.0482 (14)	−0.0036 (10)	0.0032 (10)	−0.0029 (11)
C6	0.0455 (14)	0.0326 (12)	0.0504 (14)	−0.0013 (11)	0.0070 (11)	0.0043 (11)
C7	0.0390 (13)	0.0366 (12)	0.0347 (12)	0.0090 (10)	0.0016 (10)	−0.0067 (10)

### Geometric parameters (Å, º)

**Table:**

O6—H6A	0.8500	O3—H3B	0.8500
O6—H6B	0.8502	O4—H4A	0.8500
Na1—O1	2.3632 (15)	O4—H4B	0.8500
Na1—O5^i^	2.3872 (16)	O5—Na1^i^	2.3872 (16)
Na1—O3^ii^	2.4032 (16)	O5—H5A	0.8499
Na1—O4	2.4239 (17)	O5—H5B	0.8500
Na1—O3	2.5142 (17)	C1—C2	1.524 (3)
Na1—O5	2.5187 (16)	C2—C4	1.505 (3)
Na1—Na1^i^	3.5391 (15)	C2—H2A	0.9700
Na1—Na1^ii^	3.5823 (15)	C2—H2B	0.9700
Cl1—C7	1.744 (2)	C3—C4	1.376 (3)
N1—C7	1.319 (3)	C3—H3	0.9300
N1—C3	1.344 (3)	C4—C5	1.386 (3)
O1—C1	1.246 (2)	C5—C6	1.371 (3)
O2—C1	1.271 (2)	C5—H5	0.9300
O3—Na1^ii^	2.4032 (16)	C6—C7	1.374 (3)
O3—H3A	0.8500	C6—H6	0.9300
			
H6A—O6—H6B	107.0	Na1—O3—H3B	105.2
O1—Na1—O5^i^	107.88 (6)	H3A—O3—H3B	106.9
O1—Na1—O3^ii^	95.50 (6)	Na1—O4—H4A	124.6
O5^i^—Na1—O3^ii^	88.18 (5)	Na1—O4—H4B	121.3
O1—Na1—O4	97.59 (6)	H4A—O4—H4B	108.1
O5^i^—Na1—O4	79.97 (5)	Na1^i^—O5—Na1	92.30 (5)
O3^ii^—Na1—O4	164.52 (6)	Na1^i^—O5—H5A	122.3
O1—Na1—O3	89.29 (5)	Na1—O5—H5A	99.2
O5^i^—Na1—O3	162.45 (6)	Na1^i^—O5—H5B	122.0
O3^ii^—Na1—O3	86.51 (6)	Na1—O5—H5B	107.6
O4—Na1—O3	101.86 (6)	H5A—O5—H5B	107.8
O1—Na1—O5	163.18 (6)	O1—C1—O2	125.41 (19)
O5^i^—Na1—O5	87.70 (5)	O1—C1—C2	120.11 (19)
O3^ii^—Na1—O5	78.27 (5)	O2—C1—C2	114.48 (18)
O4—Na1—O5	91.24 (6)	C4—C2—C1	118.45 (18)
O3—Na1—O5	74.85 (5)	C4—C2—H2A	107.7
O1—Na1—Na1^i^	152.66 (6)	C1—C2—H2A	107.7
O5^i^—Na1—Na1^i^	45.32 (4)	C4—C2—H2B	107.7
O3^ii^—Na1—Na1^i^	80.43 (4)	C1—C2—H2B	107.7
O4—Na1—Na1^i^	84.15 (5)	H2A—C2—H2B	107.1
O3—Na1—Na1^i^	117.20 (5)	N1—C3—C4	124.5 (2)
O5—Na1—Na1^i^	42.37 (4)	N1—C3—H3	117.8
O1—Na1—Na1^ii^	93.18 (5)	C4—C3—H3	117.8
O5^i^—Na1—Na1^ii^	130.38 (5)	C3—C4—C5	116.4 (2)
O3^ii^—Na1—Na1^ii^	44.47 (4)	C3—C4—C2	121.4 (2)
O4—Na1—Na1^ii^	142.25 (6)	C5—C4—C2	122.1 (2)
O3—Na1—Na1^ii^	42.04 (4)	C6—C5—C4	120.6 (2)
O5—Na1—Na1^ii^	71.35 (4)	C6—C5—H5	119.7
Na1^i^—Na1—Na1^ii^	102.08 (4)	C4—C5—H5	119.7
C7—N1—C3	116.48 (18)	C5—C6—C7	117.5 (2)
C1—O1—Na1	121.26 (13)	C5—C6—H6	121.2
Na1^ii^—O3—Na1	93.49 (6)	C7—C6—H6	121.2
Na1^ii^—O3—H3A	102.7	N1—C7—C6	124.4 (2)
Na1—O3—H3A	123.6	N1—C7—Cl1	115.97 (16)
Na1^ii^—O3—H3B	126.5	C6—C7—Cl1	119.60 (17)

Symmetry codes: (i) −*x*+1, −*y*+1, −*z*+1; (ii) −*x*+1, −*y*+2, −*z*+1.

### Hydrogen-bond geometry (Å, º)

**Table:**

*D*—H···*A*	*D*—H	H···*A*	*D*···*A*	*D*—H···*A*
O3—H3*A*···O4^iii^	0.85	2.06	2.893 (2)	168
O4—H4*A*···O2^iv^	0.85	1.91	2.762 (2)	175
O3—H3*B*···O6^v^	0.85	1.97	2.775 (2)	159
O4—H4*B*···O2^v^	0.85	1.98	2.824 (2)	169
O5—H5*A*···O6^v^	0.85	2.08	2.886 (2)	157
O5—H5*B*···O1^ii^	0.85	2.07	2.9214 (19)	175
O6—H6*A*···N1^vi^	0.85	2.05	2.900 (2)	173
O6—H6*B*···O2	0.85	1.95	2.796 (2)	176

Symmetry codes: (ii) −*x*+1, −*y*+2, −*z*+1; (iii) *x*, *y*+1, *z*; (iv) *x*, *y*−1, *z*; (v) −*x*+1, *y*−1/2, −*z*+1/2; (vi) −*x*+2, *y*+1/2, −*z*+1/2.
